# Etomidate versus Propofol for Electroconvulsive Therapy in Patients with Major Depressive Disorders in Terms of Clinical Responses to Treatment: A Retrospective Analysis

**DOI:** 10.3390/brainsci13071023

**Published:** 2023-07-03

**Authors:** In-Young Yoon, Jung-Hee Ryu, Sang-Hwan Do, Beomjun Min, Chang-Hoon Koo

**Affiliations:** 1 Department of Psychiatry, Seoul National University Bundang Hospital, Seongnam 13620, Republic of Korea; iyoon@snu.ac.kr; 2 Department of Psychiatry, Seoul National University College of Medicine, Seoul 03080, Republic of Korea; 3 Department of Anesthesia and Pain Medicine, Seoul National University Bundang Hospital, Seongnam 13620, Republic of Korea; jinaryu74@gmail.com (J.-H.R.); shdo@snu.ac.kr (S.-H.D.); 4 Department of Anesthesia and Pain Medicine, Seoul National University College of Medicine, Seoul 03080, Republic of Korea; 5 Chung Psychiatric Clinic, Seoul 06614, Republic of Korea; mbj0310@gmail.com

**Keywords:** etomidate, propofol, efficacy, seizure, Clinical Global Impression scale

## Abstract

General anesthetic agents may be associated with the clinical efficacy of electroconvulsive therapy (ECT), as they may influence seizure quality and duration. Hence, a retrospective study was conducted to compare the clinical effects and seizure variables of etomidate and propofol during ECT. Patients treated with ECT under anesthesia with etomidate (n = 43) or propofol (n = 12) were retrospectively analyzed. Seizure variables (seizure duration, intensity, and threshold) and hemodynamic changes during ECT were assessed and recorded. Clinical responses to treatment were evaluated using the Clinical Global Impression scale and mood at discharge after the course of ECT. Adverse effects were also recorded. The demographic characteristics were similar between the two groups. There were no significant differences in the Clinical Global Impression scale scores, mood at discharge, and adverse effects between the two groups (*p* > 0.05); however, etomidate was associated with a significantly longer motor (42.0 vs. 23.65 s, *p* < 0.001) and electroencephalogram (51.8 vs. 33.5 s, *p* < 0.001) seizure duration than propofol. In conclusion, etomidate showed more favorable seizure profiles than propofol during ECT; however, both agents (etomidate and propofol) were associated with similar clinical efficacy profiles at discharge.

## 1. Introduction

Electroconvulsive therapy (ECT) artificially induces seizures by delivering electrical stimulation through electrodes attached to the scalp. This procedure is commonly performed to alleviate the symptoms of patients with severe or refractory depressive disorders that are uncontrolled by medications. It is performed under general anesthesia to ensure patient safety and tolerability. Various anesthetic agents, such as etomidate, propofol, ketamine, and thiopental, can be used for ECT anesthesia.

Anesthetic agents may affect seizure quality [1], and in some studies, seizure duration has also been associated with the clinical efficacy of ECT [2,3]. Most anesthetic agents, including propofol, thiopental, and midazolam, have anticonvulsant properties [4,5]; etomidate has been found to reduce the seizure threshold [6] and provide significantly longer seizure duration compared to other anesthetic agents in ECT [7].

Several studies comparing the effects of anesthetic agents during ECT in patients with depressive disorders have focused on seizure-related variables rather than on clinical improvements. We hypothesized that patients administered etomidate during ECT would achieve better clinical outcomes than those administered propofol. Therefore, this retrospective analysis aimed to compare the clinical effects of etomidate and propofol based on seizure variables and hemodynamic responses in patients with depressive disorder undergoing ECT.

## 2. Materials and Methods

### 2.1. Study Design and Study Participants

In this retrospective study, patients who were diagnosed with major depressive disorder or depressive disorder and underwent ECT at Seoul National University Bundang Hospital between June 2003 and June 2019 were enrolled. The exclusion criteria were as follows: (1) patients who did not complete the course of ECT or had undergone ECT in the past 2 months; (2) patients who received both etomidate and propofol; (3) and patients who received other anesthetic agents during the course of ECT.

### 2.2. Anesthesia for ECT

All patients were monitored using pulse oximetry, electrocardiography, and noninvasive arterial pressure measurements throughout the ECT procedures. After premedication with 0.2 mg of glycopyrrolate, anesthesia was induced with 0.2–0.3 mg/kg of etomidate or 1–2 mg/kg of propofol. Succinylcholine (1 mg/kg) was used for neuromuscular blockade. Subsequently, ECT was performed using the Thymatron IV system (Somatics, LLC, Lake Bluff, IL, USA). The stimulus dosage was selected by the attending psychiatrist based on the guidelines of the institution. The stimulus charge starts from 32 mC for female patients and from 48 mC for male patients [8]. If there was no seizure or its duration was shorter than 20 s, an additional stimulation (about 50% increased energy) was adjusted according to the protocol of the guideline. In the subsequent session, the energy of the last stimulation in the previous session was chosen as the dose of the first stimulation.

### 2.3. Outcomes

This study compared the effects of etomidate and propofol on clinical outcomes using the Clinical Global Impression (CGI) scale. For all patients undergoing ECT, the CGI scale was used by experienced psychiatrists to assess the severity of the illness. The CGI consists of a 7-point Likert scale with scores as follows: 1, normal, not at all ill; 2, borderline mentally ill; 3, mildly ill; 4, moderately ill; 5, markedly ill; 6, severely ill; and 7, extremely ill. CGI analysis was conducted at the time of admission to the hospital (before ECT) and discharge from the hospital (after ECT). The CGI score was determined based on the overall clinical appearance, including symptoms, behaviors, and functional impairments.

Secondary outcomes, including mood at discharge, full or partial remission, length of hospital stay, and adverse events related to ECT, were also analyzed. Adverse events, including amnesia, headache, anxiety, and insomnia, were recorded if any of these occurred.

ECT-related variables (doses and types of anesthetics, number of ECT sessions, and stimulus charges) and seizure-related variables (motor and electroencephalogram (EEG) seizure duration) were recorded. In terms of stimulus charges and seizure durations, the total values were divided by the number of ECT sessions because different numbers of ECT sessions could produce biased results.

Hemodynamic variables during the ECT (heart rate and blood pressure) were recorded. Hemodynamic responses were defined as maximal changes during ECT from baseline hemodynamic parameters, which were measured prior to the induction of anesthesia. The mean values of hemodynamic responses were calculated and compared.

### 2.4. Statistical Analyses

All statistical analyses were performed using the Statistical Package for the Social Sciences (SPSS) software version 25.0 (SPSS Inc., Chicago, IL, USA). Continuous variables are presented as mean with standard deviation or median with interquartile range, depending on the normality of distribution. Student’s t-test or Mann–Whitney U test was used for comparison. Categorical variables are presented as numbers with percentages, and the chi-squared test or Fisher’s exact test was used for comparison. For missing values, mean values were substituted for missing values by using unconditional mean imputation.

## 3. Results

**Figure 1:**
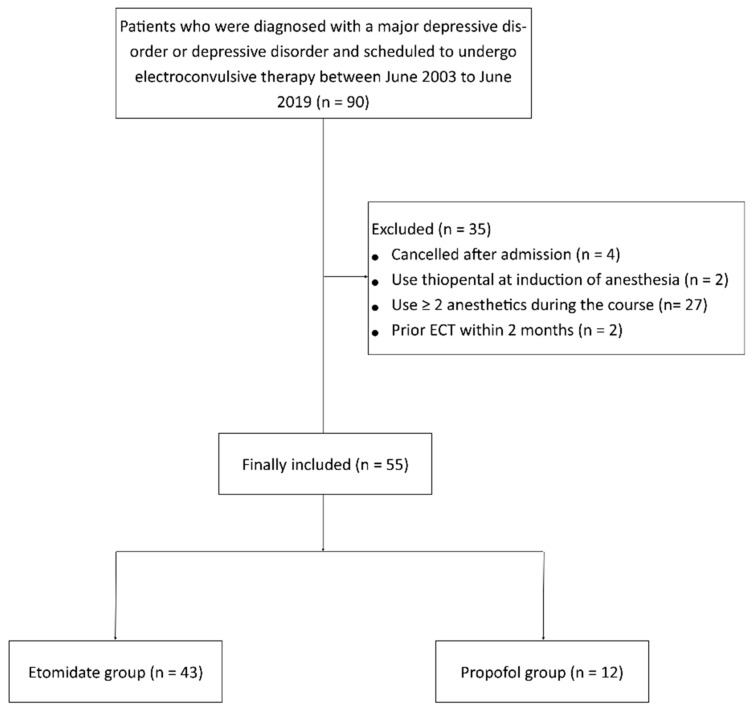
Flow diagram of included and excluded patients.

In total, 90 patients were scheduled to undergo ECT between June 2003 and June 2019. Of these, four patients were excluded from the analysis because they canceled ECT after admission. Two patients who received thiopental during anesthesia induction and 27 patients who received more than two anesthetics during the ECT course were excluded. Two patients who had undergone ECT within the last 2 months were also excluded. Hence, 55 patients were included in the final analysis, of whom 43 were administered etomidate, whereas 12 received propofol during anesthetic induction (Figure 1).

The demographic characteristics and baseline hemodynamic parameters are shown in Table 1. Based on the anesthetic administered, the patients were divided into etomidate and propofol groups.

**Figure 2:**
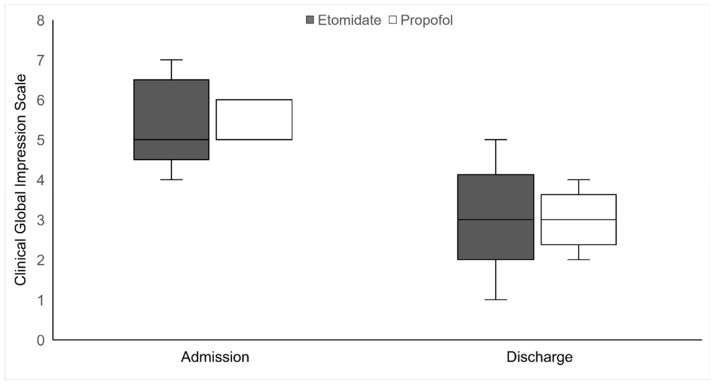
Clinical Global Impression scale scores of the two groups at admission and discharge.

The CGI scores at admission and discharge are shown in Figure 2. CGI scores at admission were not available for six patients (three in each group); thus, they were excluded from the CGI analysis.

The CGI scores were comparable between the two groups (at admission, 5 [5–6] and 6 [5–6], *p* = 0.091; at discharge, 3 [3–3.25] and 3 [2.75–3.25], *p* = 0.352).

The other clinical outcomes at discharge are summarized in Table 2. No significant differences in mood at discharge, remission, and length of hospital stay were found between the two groups. No patient in the etomidate group experienced anxiety, while two in the propofol group experienced anxiety after the ECT (*p* = 0.044), which was statistically significant.

The ECT parameters, including the number of ECT sessions, seizure duration, and stimulus charges, are described in Table 3. There were some missing data regarding seizure duration (seven patients in the etomidate group and two patients in the propofol group) and stimulus charge (one patient in the etomidate group) because of lack of records. Hence, missing data were imputed using the unconditional mean imputation. 

Significant differences were observed in ECT-related parameters between the two groups. Patients in the etomidate group received less sessions of ECT than those in the propofol group (7.6 vs. 10, *p* = 0.004). A significant difference in seizure duration and stimulus charges between the groups was seen. The motor (42.0 vs. 23.65 s, *p* < 0.001) and EEG (51.8 vs. 33.5, *p* < 0.001) seizure durations per session were significantly longer in the etomidate group than in the propofol group. The mean stimulus charge per session was significantly lower in the etomidate group than in the propofol group (211.2 vs. 394.75 mC, *p* < 0.001).

**Figure 3:**
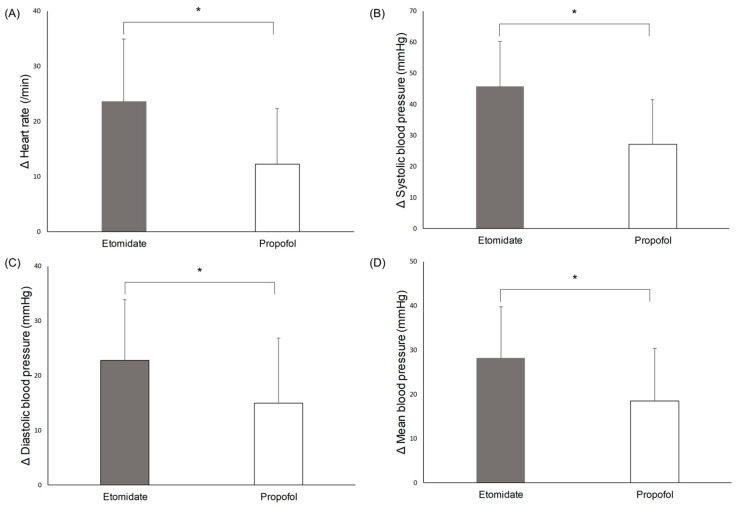
Hemodynamic changes during electroconvulsive therapy: (**A**) heart rate, (**B**) systolic blood pressure, (**C**) diastolic blood pressure, (**D**) mean blood pressure. * *p* < 0.05.

Significant hemodynamic changes during the procedure were seen in the two groups (Figure 3). The mean increase in heart rate (23.6 ± 11.3 vs. 12.3 ± 10.1 /min, *p* = 0.003), systolic blood pressure (45.7 ± 14.5 vs. 27.2 ± 14.3 mmHg, *p* < 0.001), diastolic blood pressure (22.8 ± 11.0 vs. 15 ± 11.8 mmHg, *p* = 0.036), and mean blood pressure (28.2 ± 11.6 vs. 18.5 ± 11.9 mmHg, *p* = 0.014) were significantly higher in the etomidate group than in the propofol group.

## 4. Discussion

This retrospective analysis showed that etomidate demonstrated similar clinical outcomes to propofol, despite more favorable seizure profiles (longer seizure duration and lower stimulus charge) in patients with depressive disorder undergoing ECT. Hemodynamic responses to the ECT stimulus were significantly higher in the etomidate group than in the propofol group.

To the best of our knowledge, this is the first study using the CGI scale to validate the efficacy of ECT in patients with depression. CGI is a quick, simple, and easy tool for experienced physicians to evaluate a patient’s current condition based on an overall assessment of mental status, symptoms, behaviors, and functional impairment [9]. It is a reliable tool for the evaluation of psychiatric disorder [10]. There are two types of CGI: CGI-severity (CGI-S) and CGI-improvement (CGI-I) [11]. CGI-S assesses the current severity of illness, while CGI-I evaluates the degree of change before and after treatment [12]. Both CGI types use a seven-point Likert scale. In our institution, CGI-S was routinely used and recorded when the patients were discharged, but not CGI-I. This can be attributed to patient characteristics and medical systems. Patients were hospitalized for approximately 30 days in this study, but resident physicians in charge of the medical records were rotated every month. Therefore, it seemed difficult to trace and compare the severity of illness during their hospital stay.

No significant difference in the CGI scores on discharge was seen between the two groups. These results are consistent with those of previous studies [13,14]. Eranti et al. investigated the effects of anesthetic agents on the therapeutic response to ECT [13]. The responses were classified as complete recovery, major improvement, minor improvement, no change, and worsening. The choice of anesthetic agents, including etomidate and propofol, has no influence on the response rate after ECT. Graveland et al. [14] analyzed patients with depression undergoing ECT using the Hamilton Rating Scale for Depression (HAM-D) and Montgomery–Asberg Depression Rating Scale (MADRS). No significant differences in remission and response rates and reduction in HAM-D or MADRS scores were found, regardless of the anesthetic agent used. To assess the severity of depression, the authors employed specific tools, the HAM-D and MADRS, which focused on depressive symptoms. In contrast, the CGI used in our study assesses the overall and general conditions [15]. However, CGI shows similar performance to the HAM-D and MADRS [16], although it is a more conservative tool than the HAM-D and MADRS [15].

Seizure durations were significantly longer in the etomidate group compared with the propofol group. This is consistent with previous studies, which reported that seizure duration was prolonged with etomidate compared to propofol [17,18]. Among various anesthetic agents, etomidate induces the longest seizure duration, whereas propofol induces the shortest seizure duration [19]. This concurs well with orders of anesthetic agents reducing seizure threshold and confirms our findings. Etomidate may act as a proconvulsant [20], whereas propofol may act as an anticonvulsant [21]. This maybe the reason why the stimulus charges were significantly lower in the etomidate group than in the propofol group. The findings of this study imply that seizure duration is not correlated with clinical improvement in patients with depression undergoing ECT.

It is worth noting that patients in the etomidate group significantly required less ECT sessions than those in the propofol group. It may imply patients who were administered propofol need more treatment sessions to achieve the comparable clinical improvement compared with those who were administered etomidate. However, these findings need to be interpreted with caution due to relatively smaller number of patients included in the propofol group. The difference might be a statistical bias resulting from the smaller sample size, rather than a true difference.

The increase in hemodynamic parameters, such as heart rate and blood pressure, were more prominent after etomidate administration. This is in agreement with several studies, which concluded that propofol provided hemodynamic stability in comparison to etomidate during ECT procedures [22,23,24]. ECT stimulates the sympathetic nervous system, inducing unstable hemodynamic responses, such as tachycardia and hypertension [25]. Etomidate has a limited effect on cardiovascular function, leading to unstable hemodynamic responses associated with ECT [26]. In contrast, given that cardiovascular depression is seen with propofol, the hemodynamic response to ECT can be attenuated after propofol administration [26].

### Limitations

The present study has some limitations. First, since this was a retrospective study, we could not control for all confounding factors that might have affected the clinical outcomes. Second, the selection of anesthetics was not unified during the course of the ECT in several patients. In our institution, anesthetic induction agents are selected at the discretion of anesthesiologists or psychiatrists. Consequently, a quarter of the patients were excluded from the analysis. Furthermore, etomidate was preferred due to its advantage-related seizure duration, which resulted in a difference in sample sizes between the groups. Uneven sample size could potentially lead to an over- or under-interpretation of the data, reducing the reliability of our results. Third, CGI was rated by several psychiatrists; hence, there is a possibility of inter-rater variability in the assessment of the CGI. In addition, CGI was the only scale used to evaluate the patients in this retrospective study. Future studies including additional evaluation scales, such as subjective scales, and the incorporation of metrics, like time to recovery, are needed to ensure a more detailed comparison between the impacts of etomidate and propofol. A large-scale prospective randomized clinical trial should be conducted to evaluate the clinical effects of etomidate use during ECT in patients with major psychiatric disorders.

## 5. Conclusions

This retrospective study analyzed and compared the clinical outcomes of etomidate and propofol during ECT in patients with major depressive disorders. Etomidate showed clinical outcomes similar to those of propofol, despite having more favorable seizure profiles.

## Figures and Tables

**Table 1:** Demographic data.

Variables	Etomidate (n = 43)	Propofol (n = 12)	*p* Value
Age, years	66.2 ± 11.5	63.8 ± 10.4	0.516
Female sex, n (%)	32 (74.4%)	10 (83.3%)	0.709
BMI, kg/m^2^	23.4 ± 3.6	21.7 ± 2.3	0.063
Drug treatment			
SSRI, n (%)	22 (51.2%)	11 (91.7%)	0.018 *
SNRI, n (%)	17 (39.5%)	5 (41.7%)	0.894
TCA, n (%)	15 (34.9%)	6 (50.0%)	0.341
NDRI, n (%)	6 (14.0%)	1 (8.3%)	1.000
BDZ, n (%)	34 (79.1%)	11 (91.7%)	0.430
Antipsychotics, n (%)	31 (72.1%)	10 (83.3 %)	0.709
Baseline hemodynamic parameter			
Heart rate, /min	90.4 ± 10.4	93.7 ± 10.2	0.325
Systolic blood pressure, mmHg	136.7 ± 12.8	138.0 ± 12.2	0.863
Diastolic blood pressure, mmHg	81.4 ± 9.7	79.7 ± 7.1	0.580
Mean blood pressure, mmHg	96.2 ± 9.6	97.2 ± 8.3	0.740

The data are presented as mean ± standard deviation or number (percentage). Abbreviations: BMI = body mass index; SSRI = selective serotonin reuptake inhibitor; SNRI = serotonin–norepinephrine reuptake inhibitor; TCA = tricyclic antidepressant; NDRI = norepinephrine–dopamine reuptake inhibitor; BDZ = benzodiazepine. * *p* < 0.05.

**Table 2:** Clinical outcomes at discharge.

	Etomidate (n = 43)	Propofol (n = 12)	*p* Value
Mood			0.530
Euthymic	28 (65.1%)	10 (83.3%)	
Mild depressive	12 (27.9%)	2 (16.7%)	
Moderate depressive	3 (7.0%)	0	
Remission			0.199
Full remission	6 (14.0%)	4 (33.3%)	
Partial remission	37 (86.0%)	8 (66.7%)	
Length of hospital stay	30 [24, 39]	37 [23.5, 50.5]	0.380
Side effect			
Amnesia	13 (30.2%)	4 (33.3%)	1.000
Headache	1 (2.3%)	1 (8.3%)	0.392
Anxiety	0	2 (16.7%)	0.044 *
Insomnia	0	1 (8.3%)	0.218

The data are presented as number (%) or median [interquartile range]. * *p* < 0.05.

**Table 3:** ECT parameters.

Variables	Etomidate (n = 43)	Propofol (n = 12)	*p* Value
Number of ECT	7.6 ± 2.2	10.0 ± 3.2	0.004 *
Mean motor seizure (s)	42.0 [36.8, 51.4]	23.65 [22.65, 28.5]	<0.001 *
Mean EEG seizure (s)	51.8 [44.15, 56.2]	33.5 [31.3, 37.825]	<0.001 *
Mean stimulus charge (mC)	211.2 [165.4, 286.65]	394.75 [293.5, 660.8]	0.001 *

The data are presented as number (%) or median [interquartile range]. * *p* < 0.05

## Data Availability

The datasets generated and analyzed during this study are available on request to the corresponding author on reasonable request.
